# Supplementary material for: A Pyrazolate Osmium(VI) Nitride Exhibits Anticancer Activity through Modulating Protein Homeostasis in HepG2 Cells
Source: Int J Mol Sci. 2022 Oct 24;23(21):12779. doi: 10.3390/ijms232112779 (PMC9656236; doi:10.3390/ijms232112779)
Supplement: Supplementary file 1 [file ijms-23-12779-s001.zip › ijms-1967005-supplementary.pdf]

International Journal of Molecular Science

Supplementary Information

A Pyrazolate Osmium(VI) Nitride Exhibits Anticancer Activity Through Modulating Protein Homeostasis in HepG2 Cells

Chengyang Huang[1,2], Wanqiong Huang[3], Pengchao Ji[3], Fuling Song[3], Tao Liu[3], Meiyang Li[1], Hongzhi Guo[1], Yongliang Huang[3], Cuicui Yu[1], Chuanxian Wang[3], and Wenxiu Ni[3,*]

Table of Contents

Figure S1. The ^1H NMR spectrogram of $Na[Os^{VI}(N)(tpm)_2]$ in d_6-DMSO........................ 2

Figure S2. The IR spectrogram of $Na[Os^{VI}(N)(tpm)_2]$... 2

Table S1 Crystal data and structure refinements for $Na[Os^{VI}(N)(tpm)_2]$ 3

Table S2 Selected bond lengths (Å) and angles (º) for $Na[Os^{VI}(N)(tpm)_2]$.................... 4

Table S3 The Na content of $Na[Os^{VI}(N)(tpm)_2]$ detected by ICP-MS.............................. 4

Figure S3. The ESI-MS spectra of $Na[Os^{VI}(N)(tpm)_2]$ in MeOH, acquired in negative ion mode. Insets right show the experimental (black) and simulated (red) patterns for the peak at m/z 562.. 5

Figure S4. The stability of $Na[Os^{VI}(N)(tpm)_2]$ (30μM) in DMSO and in PBS (3% DMSO). (T=298K, path length=1cm).. 5

Figure S5. The stability of $Na[Os^{VI}(N)(tpm)_2]$ (30μM) with different concentration of GSH in PBS (3% DMSO). Os represents $Na[Os^{VI}(N)(tpm)_2]$. (T=298K, path length=1cm)... 6

Figure S6. Effects of different concentrations of $Na[Os^{VI}(N)(tpm)_2]$ on the morphology of HepG2 cells at 3 h. 6

Table S4 Abbreviations in the main text.. 7

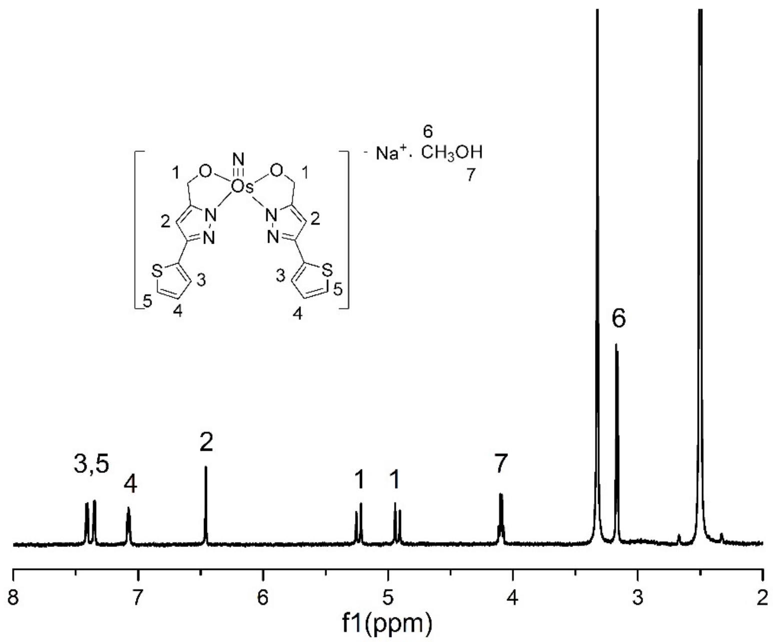

Figure S1. The ^{1}H NMR spectrogram of **Na[Os^{VI}(N)(tpm)$_2$]** in d$_6$-DMSO.

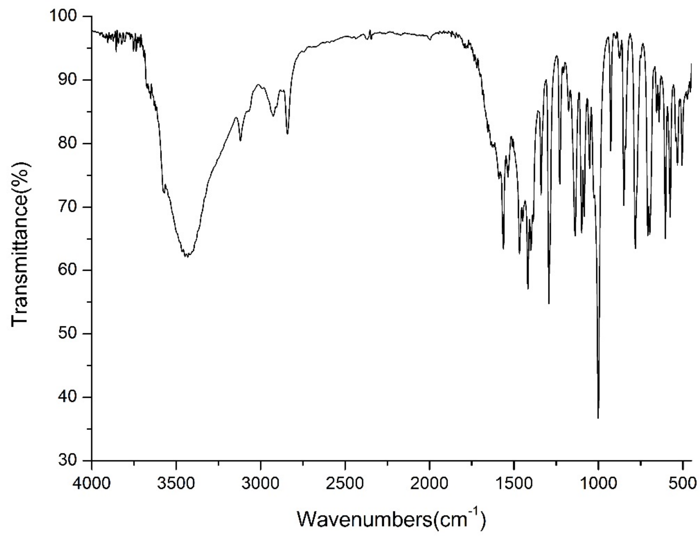

Figure S2. The IR spectrogram of **Na[Os^{VI}(N)(tpm)$_2$]**.

Table S1 Crystal data and structure refinements for **Na[Os^{VI}(N)(tpm)$_2$]**

	**Na[Os^{VI}(N)(tpm)$_2$]**
Formula	$C_{17}H_{16}N_5NaO_3OsS_2$
Mr.	615.66
Crystal system	Orthorhombic
Space group	$P\ 2_12_12_1$
Temp (K)	293(2)
a (Å)	7.52480(10)
b (Å)	13.9934(3)
c (Å)	18.9699(3)
α (°)	90
β (°)	90
γ (°)	90
V (Å^3)	1997.48(6)
Z	4
D_c (g·cm^{-3})	2.047
no. of reflns	4617
no. of unique	3330
data collected	3.93 to 74.54
unique refl.(R_{int})	0.0283
GOF on F^2	1.086
R_1[I≥2σ(I)][a]	0.0374
wR_2[I≥2σ(I)][b]	0.1018
R_1[all data]	0.0381
wR_2[all data]	0.1022
Flack parameter	0.33(2)

[a]$R_1 = \Sigma||F_o| - |F_c||/\Sigma|F_o|$. [b] $wR_2 = [\Sigma[w(F_o^2 - F_c^2)^2]/\Sigma w(F_o^2)^2]^{1/2}$, where $w = 1/[\ w^2(F_o)^2 + (aP)^2 + bP]$ and $P = (F_o^2 + 2F_c^2)/3$.

Table S2 Selected bond lengths (Å) and angles (°) for **Na[Os^{VI}(N)(tpm)$_2$]**.

Os(1)-O(1)	1.965(7)	Os(1)-O(2)	1.956(8)	Os(1)-N(1)	2.000(8)
Os(1)-N(3)	2.006(8)	Os(1)-N(5)	1.649(9)	Na(1)-O(1)#2	2.493(9)
Na(1)-O(2)#2	2.388(9)	Na(1)-O(3)	2.116(7)	Na(1)-N(2)	2.463(9)
Na(1)-N(4)	2.511(10)				
O(2)-Os(1)-O(1)	82.3(3)	O(2)-Os(1)-N(1)	143.5(4)	O(2)-Os(1)-N(3)	80.0(4)
O(1)-Os(1)-N(1)	79.5(3)	O(1)-Os(1)-N(3)	144.0(3)	N(1)-Os(1)-N(3)	96.8(3)
N(5)-Os(1)-O(2)	109.9(5)	N(5)-Os(1)-O(1)	109.3(4)	N(5)-Os(1)-N(1)	106.0(4)
N(5)-Os(1)-N(3)	106.1(4)	O(2)#2-Na(1)-O(1)#2	63.8(3)	O(2)#2-Na(1)-N(4)	109.2(3)
O(2)#2-Na(1)-N(2)	168.9(4)	O(1)#2-Na(1)-N(4)	171.3(3)	N(2)-Na(1)-O(1)#2	106.7(3)
N(2)-Na(1)-N(4)	79.7(3)	O(3)-Na(1)-O(2)#2	114.4(3)	O(3)-Na(1)-O(1)#2	115.9(3)
O(3)-Na(1)-N(4)	71.2(3)	O(3)-Na(1)-N(2)	74.4(3)		
Os(1)-O(2)-Na(1)#1	109.1(4)	Os(1)-O(1)-Na(1)#1	104.8(3)		

Symmetry transformations used to generate equivalent atoms: #1 x-1, y, z #2 x+1, y, z

Table S1 The Na content of **Na[Os^{VI}(N)(tpm)$_2$]** detected by ICP-MS.

Sample weight	Calculated content of Na（mg）	Measured content of Na (mg)
2.52mg	0.0993	0.09725
4.86mg	0.1915	0.16975

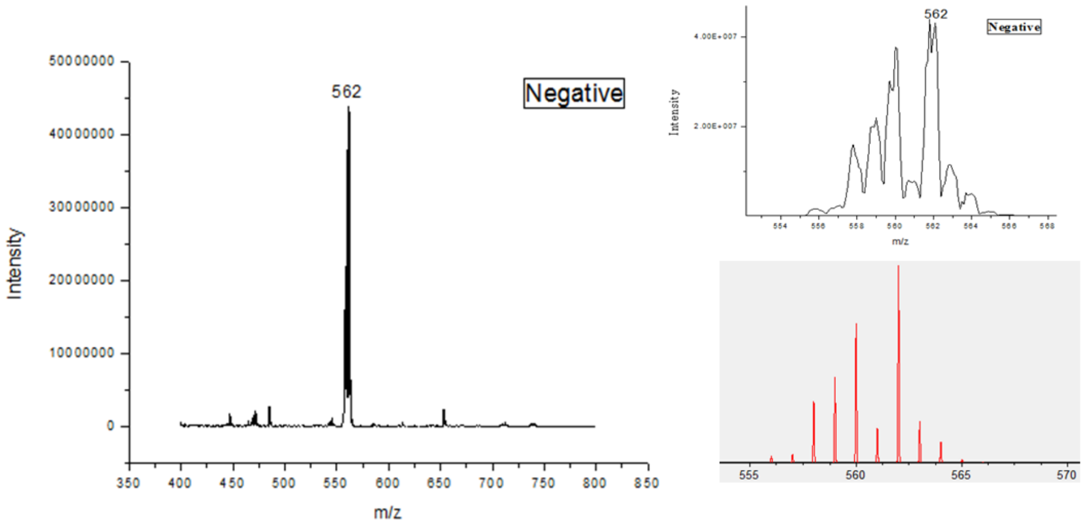

Figure S3. The ESI-MS spectra of **Na[Os^{VI}(N)(tpm)$_2$]** in MeOH, acquired in negative ion mode. Insets right show the experimental (black) and simulated (red) isotopic patterns for the peak at m/z 562.

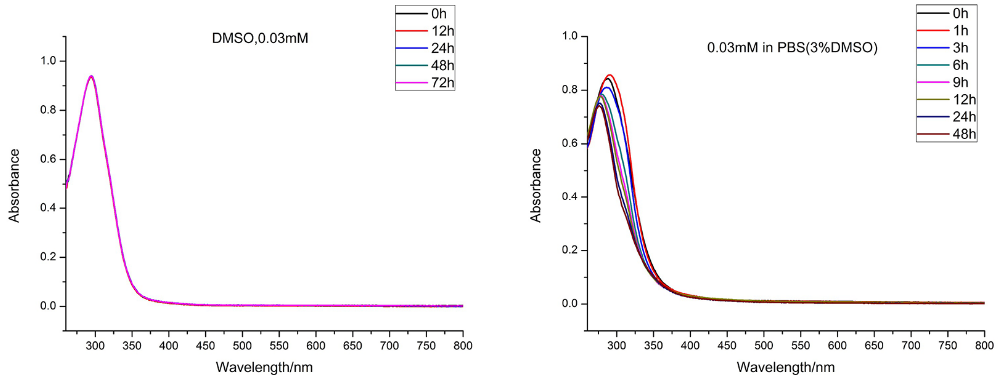

Figure S4. The stability of **Na[Os^{VI}(N)(tpm)$_2$]** (30μM) in DMSO and in PBS (3% DMSO). (T=298K, path length=1cm)

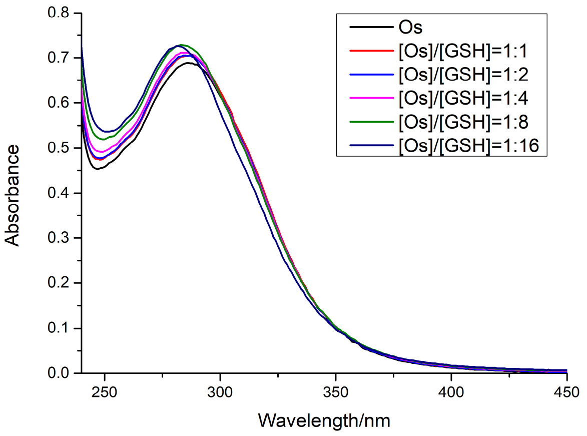

Figure S5. The stability of **Na[Os^{VI}(N)(tpm)$_2$]** (30μM) with different concentration of GSH in PBS (3% DMSO). Os represents **Na[Os^{VI}(N)(tpm)$_2$]**. (T=298K, path length=1cm)

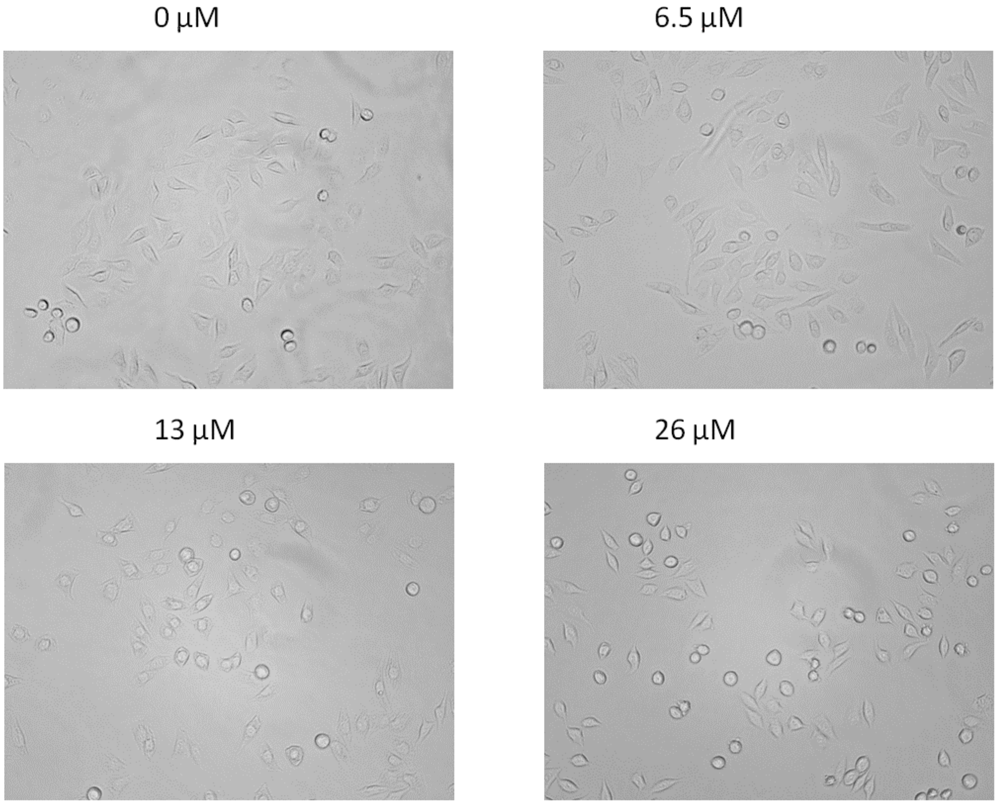

Figure S6. Effects of different concentrations of **Na[Os^{VI}(N)(tpm)$_2$]** on the morphology of HepG2 cells at 3 h.

Table S4 Abbreviations in the main text.

Abbreviations	Explanations
INTS3	Integrator Complex Subunit 3
hSSB1	human ssDNA binding protein SSB1
SMARCA1	SWI/SNF Related, Matrix Associated, Actin Dependent Regulator Of Chromatin, Subfamily A, Member 1
DDB	Damage Specific DNA Binding Protein 2
TYMS	Thymidylate Synthetase
HCC	hepatic cell carcinoma
TOP2A	topoisomerase II α
NER	Nucleotide Excision Repair
PAQR3	Progestin And AdipoQ Receptor Family Member 3
LRH-1	Liver receptor homolog-1
LSF/TFCP2	Transcription factor CP2
GCLM	Glutamate-Cysteine Ligase Modifier Subunit
GCLC	glutamate-cysteine ligase catalytic subunit
EPRE	electrophile response element
KYNU	Kynureninase or L-kynurenine hydrolase
PLP	Pyridoxal5-phosphatemonohydrate
Kyn	kynurenine
Ant	anthranilic acid
CCCP	carbonyl cyanide m-chlorophenylhydrazone
DCFH-DA	2’-7’dichlorofluorescin diacetate
ROS	Reactive oxygen species
MMP	Mitochondrial membrane potential
JC-1	5,5’,6,6’-tetrachloro-1,1’-3,3’-tetraethyl-benzimidazolylcarbocyanine iodide
GSH	glutathione
caspase	cysteinyl aspartate specific proteinase
PARP1	poly-(ADP-ribose)-polymerase-1
Cl-PARP1	Cleaved poly-(ADP-ribose)-polymerase-1
Cl-caspase 3	Cleaved cysteinyl aspartate specific proteinase 3
Cl-caspase 9	Cleaved cysteinyl aspartate specific proteinase 9
p-CDC2	Phosphorylation cell division cycle 2
